# A State-of-the-Art Self-Cleaning System Using Thermomechanical Effect in Shape Memory Alloy for Smart Photovoltaic Applications

**DOI:** 10.3390/ma15165704

**Published:** 2022-08-18

**Authors:** Nasir Ghazi Hariri, Ibrahim Khalil Almadani, Ibrahim Sufian Osman

**Affiliations:** Department of Mechanical and Energy Engineering, College of Engineering, Imam Abdulrahman Bin Faisal University, P.O. Box 1982, Dammam 31441, Saudi Arabia

**Keywords:** photovoltaic energy, dust accumulation, PV efficiency, thermomechanical, shape memory alloys, PV cleaning system, passive system, soiling effect

## Abstract

This research aims to present a state-of-the-art cleaning technology solution that effectively overcomes the dust accumulation issue for conventional photovoltaic systems. Although continuous innovations and advanced developments within renewable energy technologies have shown steady improvements over the past years, the dust accumulation issue remains one of the main factors hindering their efficiency and degradation rate. By harvesting abundant solar thermal energy, the presented self-cleaning system uses a unique thermomechanical property of Shape Memory Alloys to operate a solar-based thermomechanical actuator. Therefore, this study carries out different numerical and experimental validation tests to highlight the promising practicability of the developed self-cleaning system from thermal and mechanical perspectives. The results showed that the system has a life expectancy of over 20 years, which is closely equivalent to the life expectancy of conventional photovoltaic modules while operating under actual weather conditions in Dammam city. Additionally, the thermal to mechanical energy conversion efficiency reached 19.15% while providing average cleaning effectiveness of about 95%. The presented outcomes of this study add to the body of knowledge an innovative methodology for a unique solar-based self-cleaning system aimed toward smart and modern photovoltaic applications.

## 1. Introduction

The world’s awareness of renewable energy’s importance and advantages arises as the destructive impact of fossil fuels becomes a severe environmental issue. Harming fossil fuels to the environment includes global warming and greenhouse emissions, affecting the overall environmental quality. The Kingdom of Saudi Arabia (KSA) has taken serious steps intending to shift toward renewable energy as an alternative to conventional energy production methods, including fossil fuels, and aspires to achieve a 50% dependence on renewable energy by 2050 [1,2,3]. Although many renewable energy sources are available, solar PV energy shines the most. The abundant amount of energy provided via the sun is estimated to be 8000 times higher than the global energy consumption [4,5,6]. However, the efficiency of PV modules is a critical concern since the conventional PV modules’ efficiency can be affected by many factors; one of the main factors is the dust accumulation issue (DAI) [4,5]. The DAI is especially critical in areas with high dust intensity, such as the Middle East and North Africa (MENA).

The soiling effect is one of the main obstacles for PV systems, especially for areas with excessive dust accumulation rates, such as the MENA region, since such regions have high dust intensity. There are many factors affecting dust accumulation; a study by GHOLAMI et al. [7] showed that humidity, adhesion force, rain rate, the cover glass of the panel, wind speed and direction, and gravity affect the dust accumulation over a solar panel. Many research efforts have highlighted the significant reduction in efficiency due to soiling on PV modules [8,9] since soiling develops a layer on the outer surface of the PV module’s glass, reducing the light transmissivity [10]. Furthermore, the soiling layer developed on the glass’ outer surface leads to degradation effects which shorten the life span of the solar panels [10,11]. Kazem et al. [12] have demonstrated the effect of dust accumulation on the power of PV modules, where power loss due to soiling effects of up to 80% per month and 1% per day were recorded. In addition, the study suggested using some cleaning methods to eliminate the soiling effect.

In Farrokhi Derakhshandeh et al. [13] review, different cleaning systems for solar panels were compared. Although the review studied different active and passive solar cleaning systems, none of the studied passive cleaning systems can be achieved without human intersection. Deb and Brahmbhatt [14] reviewed and compared multiple studies on cleaning solar panels and proposed an automated water-free cleaning method using cleaning brushes. The cleaning system proposed showed a cleaning efficiency of about 9.05% and an effective cleaning efficiency considering the cost of the cleaning system of 6.31%. Alghamdi et al. [15] made three mechanical cleaning systems platforms to study the feasibility of each system in the Middle East region, where rainfall is lacking. The three platforms were an air-jet, vibrator, combination of the previous, and waterjet based. The results showed that waterjet cleaning increased the output power by about 27% and was considered the most efficient cleaning method in the experiment, while module vibration and air-jet reduced the soling in such a less practical matter. Alnaser et al. [16] experimented by comparing the performance of artificial cleaning with natural cleaning. Two sets of eight PV modules were used in the experiment, where the first set represented artificial cleaning while the second set represented natural cleaning. The experiment found that the first set produced an electrical power of about 1.06 KWh more than the producer’s expected power, representing a 16% increase from the expected power. On the other hand, the second set produced an electrical power of 0.97 KWh, which is 5% more than the producer’s anticipation. In 15 months, the production of dirty PV modules is expected to be 9% less than that of clean PV modules. Yadav et al. [17] proposed an automated cleaning system using rubber wipers powered by a direct current servo motor. The experimental outcome of the proposed system’s cleaning efficiency was recorded as 97.8%.

Cleaning frequency can differ in each country depending on the climate and energy cost [18]. The optimal cleaning frequency is about 25 days; for tropical regions, the cleaning frequency is less than 10 days [19]. In order to determine the optimal cleaning frequency, the overall cleaning cost must be investigated, which depends on the climate conditions, water consumption, and electrical usage [20].

In 1932, Shape Memory Alloys (SMA) was discovered, which is a type of smart material with the property to regain a programmed shape after exposure to heat called the Shape Memory Effect (SME) [21,22]. Several alloys have SME, each with distinctive characteristics, including activation temperature and yield strength. Various materials are used to make different SMAs, such as Cu-Zn-Al, Cu-Al-Ni, Au-Cd, and TiNi-Ag [23,24]. The most used type of SMA is Nickel-Titanium alloy, also known as NiTiNOL, due to its wide range of activation temperature and low price. SMA has multiple applications in various fields, including aerospace, biomedical, robotics, aeronautics, and engineering [25,26,27]. The utilization of SMA in multiple applications requires the ability to control the alloys as needed, which is possible through various techniques [28,29]. The wide range of characteristics and ability to manipulate the SMA has led to various innovative applications. QADER et al. [30] reviewed how different parameters such as stress, strain, and temperature affect SMA behavior. The review compared typical material and different SMA deformation under different stresses and strains. Additionally, the review compares steal and NiTiNOL SMA strain recovery percent and the differences in the materials’ elasticity. In addition, the review reveals how SMA behavior changes under various temperatures. Another review by Farber et al. [31] discusses the manufacturing of NiTiNOL using 3D printing techniques. The review shows how the Nickel/Titanium ratio affects the SMA’s activation temperature and shape memory effect. Such reviews facilitate a better understanding of SMA, helping further studies to better implement SMA in various applications spatially as actuators. In addition, a recent exciting study has discussed advanced technology to obtain shape memory alloy thin layer utilizing pulsed laser deposition (PLD) process [32].

Multiple studies and reviews discuss the utilization of SMA in actuation techniques for different applications. A review by Yuan et al. [33] shows different SMA-based rotary actuators’ designs. The actuators mentioned in the review were categorized depending on two features movement continuity and whether the motion is single rotation direction or bi-directional rotation. Depending on the freedom and continuity of motion and other actuator attributes, the actuation mechanism’s application differs. A review by Costanza and Tata [34] discusses the recent utilization of SMA in aerospace and aeronautics. The review shows that SMA reduces noise, increases thrust, and optimizes the efficiency of wing morphing and propulsion system. As for SMA applications in aerospace, the review shows that SMA is used for “isolating the micro-vibrations, for low-shock release devices, and self-deployable solar sails.” All of these studies demonstrate how SMA is being recognized for actuation applications in various fields.

SMA actuators can be activated via joule heating, external/internal passive, or forced heating [35]. Likewise, the offered method to activate the SMA-based actuator in this paper is via solar heat collector (SHC). SHC is a unique heat exchanger device that absorbs the natural sun rays as thermal energy input, converting it into another useful form of energy [36,37]. Solar thermal energy is more efficient than solar photovoltaic energy by about 77% [2,38,39], and many researchers have suggested different technologies to enhance its thermal efficiency [40]. SHC applications are varied, including industrial heating or cooling, energy storage systems, and water desalination systems [41,42]. SHCs have different types based on various categories, where they can be divided based on their designed shape, derived method, and the utilized fluid [36,43]. Additionally, multiple materials are used to construct the SHC absorber, such as aluminum and copper [36,40,44]. At the same time, multiple fluids are also used inside the SHC, such as water, nanofluids, and air [1,45]. Designing the SHC is a critical step in any thermal energy system, where three main criteria should be considered: technical, cost, and environmental consecrations [46]. The technical aspect focuses on the efficiency of the SHC. In contrast, the cost aspect estimates the system’s overall cost, while the environmental aspect predicts the effect of the SHC on the environment [2,47,48].

In the literature and previous studies, minimum research discussed the utilization of the natural sun rays to run thermomechanical or SMA-based actuators; however, previous research efforts by the authors have highlighted its applicability. Osman and Hariri [2] presented a detailed thermal study for a novel thermomechanical actuator. The results conclude that the system can operate under the actual weather condition of the studied area. Similarly, Almadani et al. [1] offered a mechanical study for a similar thermomechanical actuator. The output force and displacement of the actuator showed significant advantages, which revealed its great feasibility as a solar-based actuator.

This research effort aims to offer a novel design of a PV cleaning system for DAI that utilizes the unique property of SMA. Additionally, the paper presents different feasibility tests for the suggested technology solution, including numerical and practical experiments. The study is unique in its approach since the proposed PV cleaning system uses the available sun rays to operate in an innovative mechanism while eliminating human intervention and electrical storage configurations. Furthermore, the study offers a unique technology solution to overcome a critical issue within the renewable energy field, which increases the encouragement toward the transition to green, smart, and clean cities across the globe.

## 2. Materials and Methods

**Figure 1:**
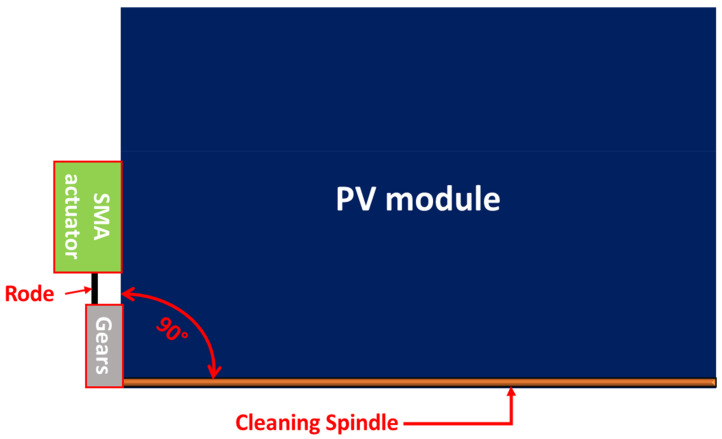
Conceptual design model of the cleaning system.

The proposed PV cleaning system contains three main parts: the cleaning spindle, gear mechanism, and thermomechanical SMA actuator assembly, as shown in Figure 1. For the working principle of the proposed design, the thermomechanical SMA actuator transforms thermal energy into mechanical energy in the form of a linear motion. Then, linear movement is converted into rotational movement through a gear mechanism, thus, allowing the cleaning spindle to slide over the PV module’s surface, which helps remove the accumulated dust particles. Therefore, this section discusses the conceptual design of the thermomechanical actuator and highlights the evaluated numerical thermal study applied. After that, it presents the development of the thermomechanical cleaning system and the evaluated mechanical assessment tests.

### 2.1. Conceptual Designs of the Thermomechanical Actuators

**Figure 2:**
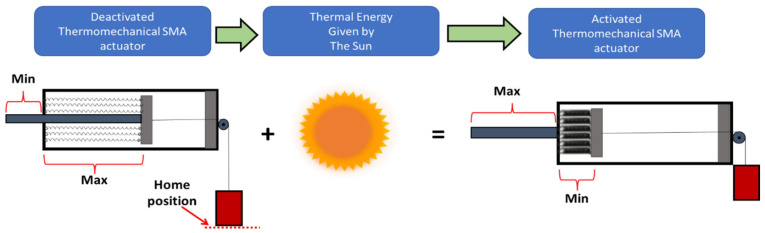
Basic conceptual procedures of the thermomechanical solar-based linear actuator.

The SMA-based actuator uses the sun’s thermal energy to give a smart movement and produces considerable force via the actuator. The actuator is typically deactivated at night-time while activated during the daytime using the sun’s rays and when it gains sufficient thermal energy from the sun, as shown in Figure 2. Therefore, the SHC supplied heat to the actuator continuously throughout the day in order to give the spring-based NiTiNOL actuator enough thermal activation energy. The actuator continues receiving solar radiation even after reaching the desired activation temperature, which becomes essential to ensure adequate activation temperature throughout the year since temperatures of ambient and actuator vary significantly. On the contrary, the actuator is typically deactivated at night-time due to the absence of the sun’s rays. The primary purpose of using a bias-load is to retain the NiTiNOL springs to their initial shape at night-time, thus, completing a single cycle daily.

**Figure 3:**
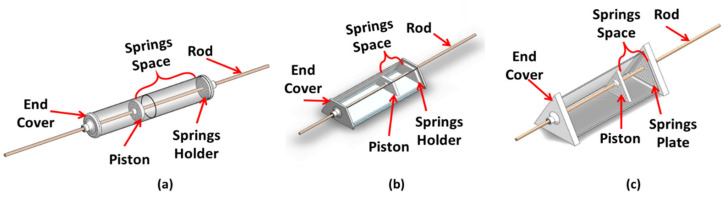
Overview of developed CAD models for (**a**) actuator A, (**b**) actuator B, and (**c**) actuator C.

The proposed thermomechanical SMA piston-based actuator consists of five main components: SHC, piston, rod, bias-load, and NiTiNOL springs. On the other hand, the actuation mechanism includes five main phases, wherein the first phase, the NiTiNOL springs, are under the austenite starting temperature (As) and extended while the bias load is at the home position. Secondly, the NiTiNOL springs absorb adequate heat to reach (As) and start contracting to pull the bias load. In the third phase, the NiTiNOL springs arrive at the austenite final temperature (Af), allowing them to reach the maximum deflection under the applied load. Fourthly, the NiTiNOL springs dissipate the heat causing the spring to reach martensite start temperature (Ms) and extend as the bias load pulls the piston. In the final phase, the NiTiNOL springs are in the (Mf), letting the springs return to the original extended shape. Multiple Computer-Aided Designs (CAD) of SHCs devices were designed and evaluated to optimize the design with the aim of completing one actuation cycle per day throughout the year. As the heat collectors change in size and shape, all components inside the heat collector change accordingly. Therefore, the first actuator design (actuator A) has a glass cylindrical heat collector, as shown in Figure 3a. The Second actuator design (actuator B) has a glass-covered aluminum isosceles trapezoid prism heat collector, as shown in Figure 3b. The third actuator design (actuator C) combines glass and aluminum for the equilateral triangle prism heat collectors, as shown in Figure 3c.

### 2.2. Numerical Study Setup of the Thermomechanical Actuators

Since the proposed system is entirely dependent on thermal energy to operate, a thermal investigation of the system becomes crucial. Therefore, a time-dependent three-dimensional (3D) thermal simulation study has been conducted for the proposed designs under the weather conditions of Dammam city, KSA using Computational Fluid Dynamics (CFD) software version 5.6. The three developed designs were imported to CFD software, where different studies have been conducted, and their results are presented in upcoming Section 3. Various boundary conditions were applied, knowing that the SHC gains thermal energy via the sun’s radiation and convection heat transfer processes. Due to the SHC design, the applied amount of heat is transferred to the NiTiNOL springs by the radiation and convection heat transfer processes. Therefore, the stated boundary conditions were applied as heat flux, located at different locations depending on the state heat transfer process. In addition, an external heat source that represents the actual sun rays of Dammam city was also included in the thermal studies as boundary conditions. Additionally, the initial conditions have been chosen to simulate the actual weather conditions of the studied area. Eventually, fine mesh and time steps for each designed model and iteration were adopted.

### 2.3. Development of a State-of-the-Art PV Cleaning System

**Figure 4:**
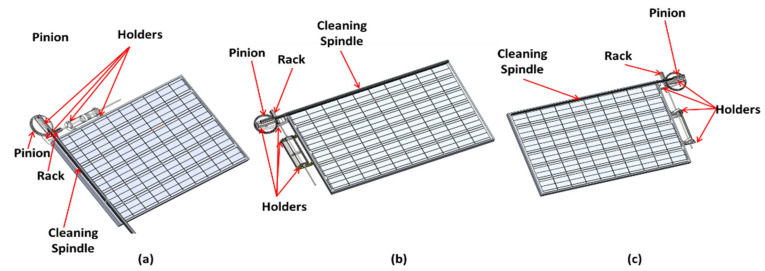
Detailed CAD model of the PV cleaning system based on (**a**) Actuator A, (**b**) Actuator B, and (**c**) Actuator C.

The proposed solar-driven smart PV modules cleaning system offers a novel solution to the dust accumulation dilemma, which varies depending on the weather and the location in which PV modules are installed. The design criteria of the smart cleaning system are meant to be portable, concise, and easy to install in order to facilitate the integration into existing solar arrays. The novel smart cleaning system consists of four main components: the thermomechanical SMA actuator, power transmission mechanism, cleaning spindle, and PV connectors. The mechanism used to transmit power from the thermomechanical SMA actuator into the cleaning spindle is a rack and pinion mechanism, where the rack is connected to the thermomechanical SMA actuator’s rod mounted into the PV module’s frame by a PV connector. In addition, the pinion is attached to a PV connector and mounted into the PV module’s frame. As for the cleaning spindle, the spindle is mounted into the pinion using the cleaning spindle holder. Figure 4 represents all components within the smart cleaning system.

The force transmission unit has been designed according to the desired performance of the smart cleaning system, where a 150 mm linear motion is converted into a 90° rotational motion. In order to do so, a rack and pinion force transmission mechanism is designed and fabricated, where the linear travel distance of the rack is known and is used to calculate the required pinion circular pitch diameter (*Dp*) as in Equation (1).

(1) S=θ∗r

where, *S* is the travel distance of the linear actuator, θ is the rotation angle of the cleaning spindle, and *r* is the radius of the pinion gear that is also equal to *Dp*. Multiple design iterations were computed to determine the optimized design and number of teeth (*T_1_*) required for the pinion gear to achieve the desired motion for the intended cleaning system. For that, *T*_1_ is computed as shown in Equations (2)–(5), respectively, where $P_{d}$ is the pitch diameter, m is the number of module, $a_{w}$ is the addendum, and $d_{w}$ is the dedendum of the gear mechanism.

(2) $$P_{d}=\frac{T_{1}}{D_{p}}$$

(3) $$m=\frac{D_{p}}{T_{1}}$$

(4) $$a_{w}=m$$

(5) $$d_{w}=1.2m$$

**Figure 5:**
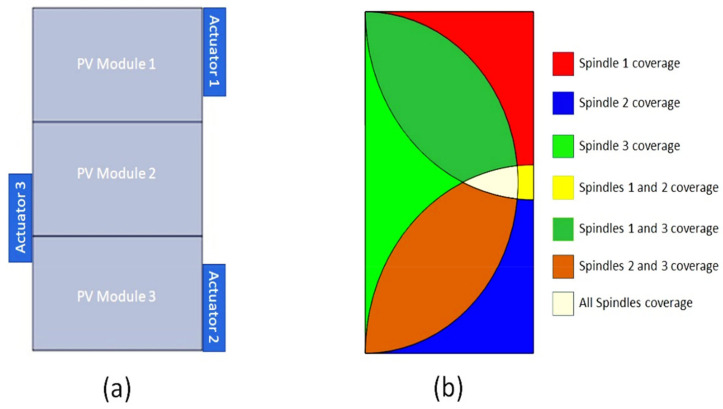
(**a**) Arbitrary CAD example of dusty PV modules string, and (**b**) coverage area of multiple actuator arrangements used for cleaning.

The following design condition shown in Equation (6) was considered for selecting *T_1_* of the pinion gear to avoid potential interference and validate the design [49].

(6) $$T_{1}\geq \frac{2a_{w}\frac{1}{T_{2}}P_{d}}{\sqrt{1+\frac{1}{T_{2}}\left(\frac{1}{T_{2}}+2\right)\sin^{2}\varphi }-1}$$

where, *T*_2_ value is set to be quarter of *T*_1_ since the required gear rotation is 90°. In addition, φ is the pressure angle with a value of 20°. Furthermore, the coverage area of the cleaning system is another critical design element for the feasibility of the cleaning system. Since most solar systems consist of multiple PV solar arrays, the cleaning system was designed to meet the needs of more than a single PV module. This was achieved by adequate arrangements of the actuator’s installed location, as seen in Figure 5. Since the designed actuation mechanism is installed on the outer frame of the PV module, this adds several advantages from a practical perspective. This includes improved accessibility and placement of the PV cleaning assembly as well as implementations of actuator arrangements for multiple PV arrays, even for already existing PV farms.

**Figure 6:**
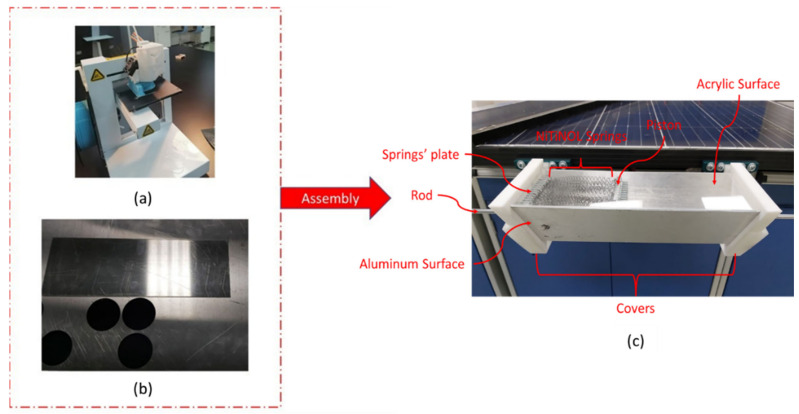
Implemented rapid prototyping techniques with (**a**) 3D printer, (**b**) laser cutting machine, and (**c**) fully assembled actuator.

The fabrication of the thermomechanical actuator involves various steps, as shown in Figure 6. It consists of two aluminum surfaces, an acrylic sheet, a piston, a springs’ plate, and a rod. In order to fabricate all different parts, several rapid manufacturing techniques were used, including laser cutting and 3D printing. In addition, an aluminum sheet was utilized to support the actuator’s structure against the axial forces that can cause bending or buckling of the actuator’s structure. It also acts as a reflecting element of the applied solar rays, which helps increase the temperature within the actuator.

**Figure 7:**
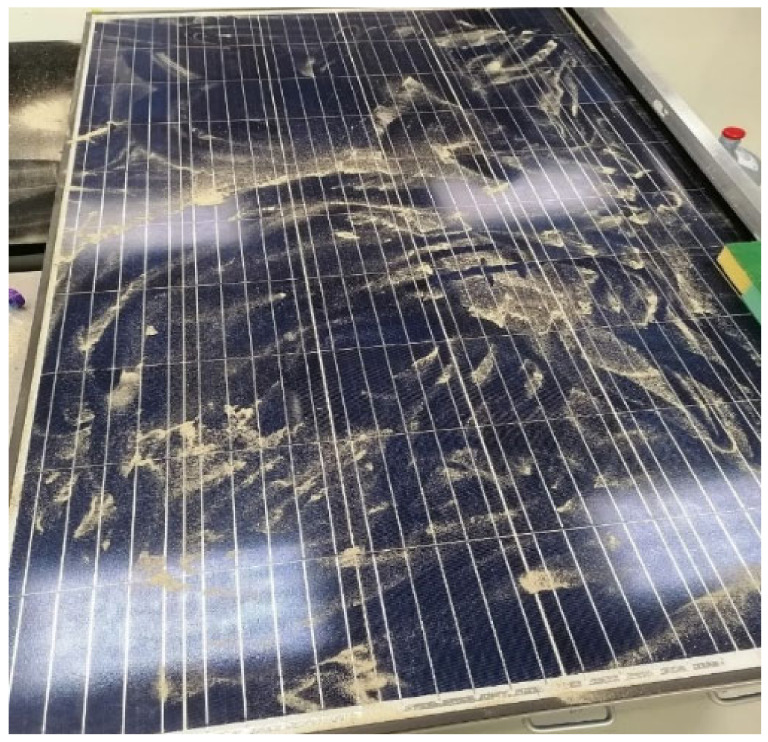
A prepared uncleaned PV module for the cleaning effectiveness test.

In order to test the cleaning effectiveness of the smart cleaning system, indoor experiments have been carried out, where a heat source, in the form of a hot air gun, was applied to activate the actuation mechanism efficiently. Thus, it allows rotating the cleaning spindle over the PV module’s top surface, which removes the accumulated dust particles. Different amounts of dust were placed over the PV module, as seen in Figure 7, to investigate the cleaning effectiveness versus dust densities.

### 2.4. Mechanical Assessment Setup of the Thermomechanical Actuator

**Figure 8:**
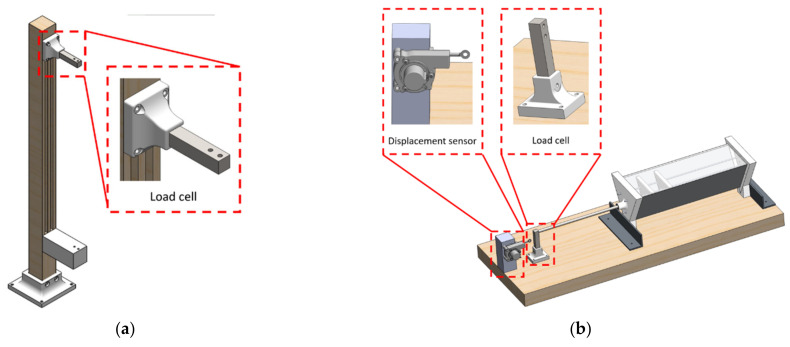
Mechanical models of the assessment platforms for the (**a**) SMA spring, and (**b**) actuator’s assembly tests.

This section discusses elements of all assessment platforms, including the developed mechanical model, electrical components, software setup, and experimental criteria. In order to carry out the tests for both the NiTiNOL springs and the actuator, two assessment platforms were built. The first test platform, seen in Figure 8a, was designed to test the generated force and displacement of the NiTiNOL springs. An active-controlled system, in the form of a gain scheduling proportional–integral–derivative (PID) controller, was implemented to quantitatively study the SMA spring-based actuator’s life expectancy. In addition, assessment tests of both displacement and force produced by the actuator’s assembly versus temperature inside the actuator were studied, as seen in Figure 8b.

**Figure 9:**
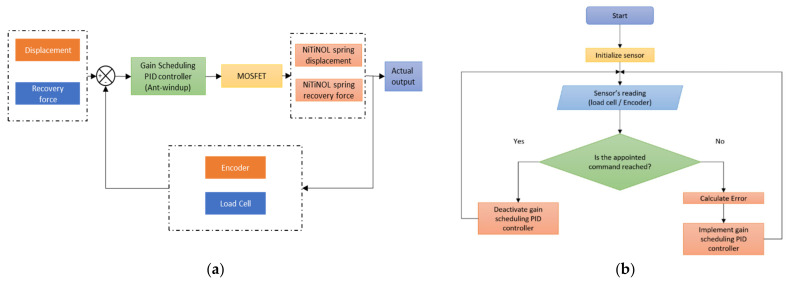
(**a**) Block diagram of the process gain scheduling PID controlled system, and (**b**) flow chart diagram of the controller working principle.

The two designed assessment platforms utilized various sensors to measure all parameters connected to a microcontroller for data collection. An accurate rotary encoder of 2000 pulses per revolution was utilized to measure the displacement produced by the actuator. In addition, a precise load cell was used for measuring the generated force, while a temperature, model LM35, was used for the temperature data. All these sensors were calibrated and programmed; a designed gain-scheduling PID controller was integrated to test the NiTiNOL springs’ life expectancy. Figure 9a shows the overall block diagram of the PID-based controller, while Figure 9b represents the flow chart diagram of the developed system.

It can be observed that each experimental test has its experimental procedure. For instance, the first assessment platform of the NiTiNOL spring force test has one side of the spring connected to the load cell while the other side was connected to a fixed hook. As the applied current passes through the spring via the Joule heating effect, heat is developed within the SMA spring, producing a recovery force. Similarly, the load cell was replaced with the rotary encoder to measure the produced motion for the displacement test. On the other hand, the applied tests for the actuation mechanism of the complete actuator assembly were performed with the replacement of the NiTiNOL spring with the actuator while reading the temperature inside the actuator using a temperature sensor.

## 3. Results and Discussion

The proposed self-operating solar-driven PV cleaning system offers a novel solution to the dust accumulation dilemma, which varies depending on the weather and the location in which PV modules are installed. Therefore, different tests were performed to validate its functionality, and their results are presented in this section. First, Section 3.1 discusses the structural analysis of the designed solar-based actuator, while Section 3.2 highlights the thermal analysis of the thermomechanical system. In addition, Section 3.3 presents the dynamic analysis of the generated forces and displacement of the actuator, while Section 3.4 highlights the overall performance of the self-cleaning PV system.

### 3.1. Structural Analysis of the Thermomechanical Actuator

**Figure 10:**
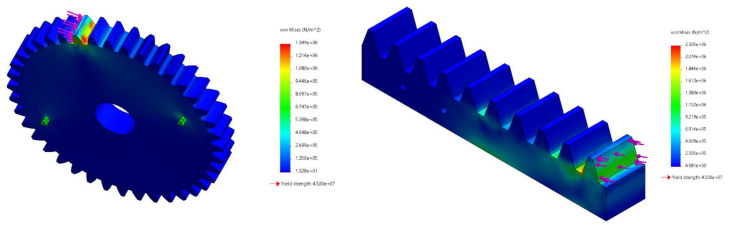
Maximum stresses developed within rack and pinion gear arrangement.

All main parts were simulated under the expected applied load to ensure the mechanical feasibility of the designed smart cleaning system. Several structural Finite Element Analysis (FEA) tests were made to avoid failure or fracture within the system’s components. The FEA results have predicted locations of critical regions where expected maximum deformations or stresses might occur. For instance, Figure 10 demonstrates an example of the maximum stresses expected under an applied load of 150 N, where the yield strength of the chosen materials (acrylic: 4.5 × 10^7^ N/m^2^, ABS: 3.9 × 10^7^ N/m^2^). Therefore, calculations for the factor of safety of all parts within the actuator assembly were computed as seen in Table 1. It can be seen that the safety factor for all parts is above one for a maximum expected load of 150 N.

### 3.2. Thermal Behavior of the Thermomechanical Actuator

**Figure 11:**
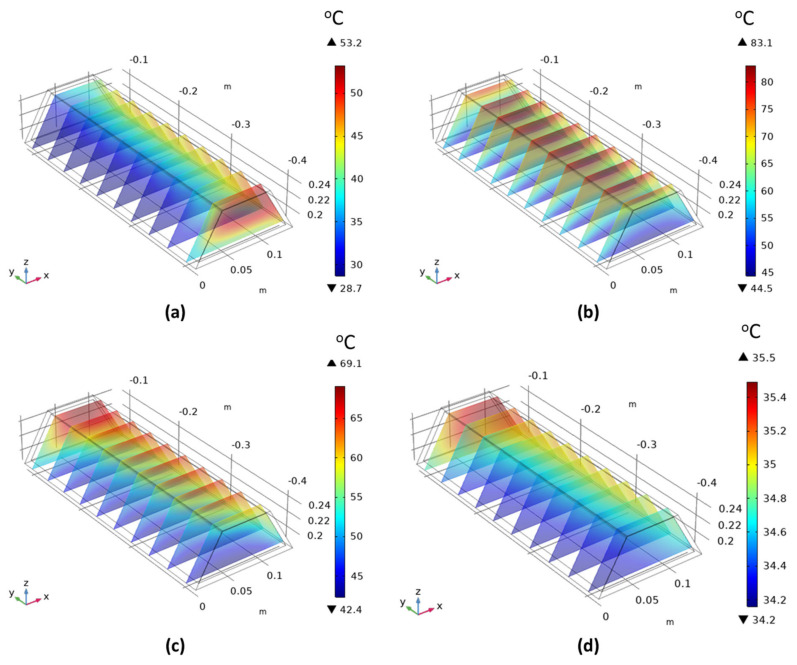
Example of 1-day temperature distributions for the designed SHC through (**a**) morning, (**b**) zenith, (**c**) afternoon, and (**d**) night periods.

Another fundamental analysis for the design of the thermomechanical actuator is the thermal performance of the designed SHC, which includes the thermal distribution and profile throughout the year. Figure 11 presents an example of a 1-day temperature distribution of the designed SHC of actuator B at different day times. It is evident in Figure 11a that the temperature on the east side (− *y*-axis) has received the highest temperature of about 52 °C due to the sun’s location in the morning. Alternatively, the temperature distribution inside the SHC is identical through the SHC length at the zenith time and when the sun is vertically toward the system, as shown in Figure 11b. It can be noticed that the maximum temperature of about 81 °C within the SHC was obtained during the zenith period. On the other hand, the west direction (+ *y*-axis) absorbs the most heat after noontime with a maximum temperature above 65 °C, as seen in Figure 11c. Lastly, the temperature distribution at late night times is shown in Figure 11d, reaching its minimum values during the day as expected.

**Figure 12:**
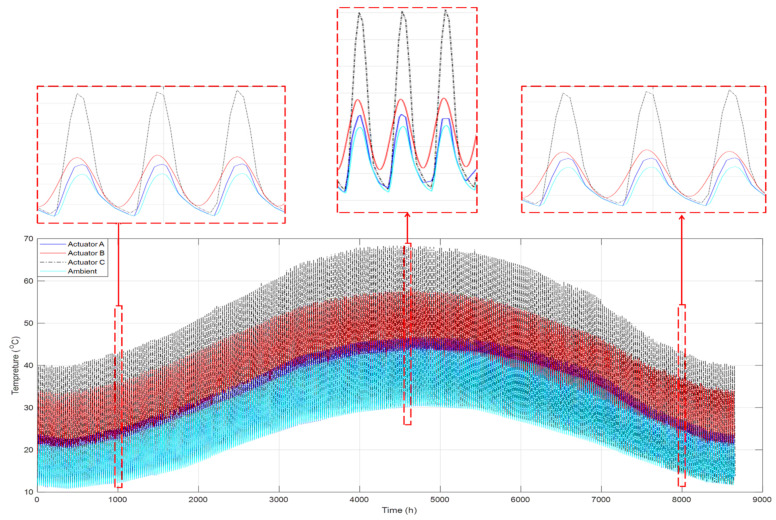
Temperature profiles inside the SHC over an entire year.

Furthermore, an extended quantitative analysis of temperature profiles for the designed SHC devices under actual weather conditions has been studied for an entire year. This allows verifying the functionality of the proposed actuators as standalone smart thermomechanical devices. As a result, the yearly temperature profiles for the three designed actuators mentioned earlier were computed, as shown in Figure 12. It can be seen that the optimized design was found to be actuator C, which shows the highest temperature profile and, most importantly, variation throughout the year. Overall, the obtained results show the ability of each SHC to increase the temperature, where actuators C, B, and A were the most efficient, respectively. The optimized design of actuator C increases the internal temperature by almost double the ambient temperature of the studied region. However, actuator B increases the temperature by about 70%, while Actuator A only increases the temperature by about 5%. The effect of the ambient temperature profile can also be seen in governing the temperature inside all designed SHCs; additionally, obtained results from this graph show that the deactivation levels are almost similar throughout the year, which is bounded by the ambient temperature during the evening time.

In addition, Table 2 shows statistically averaged data for the temperature profile inside the SHC over one year. These results have also concluded that actuator C has the highest maximum, range, and standard deviation, while actuator B has the highest mean and median values; additionally, actuator A has the lower average temperature. Hence, the highlighted statistical outcome supports that actuator C is the most efficient and adequate actuator for the studied area since it has the highest range and standard deviation values.

**Figure 13:**
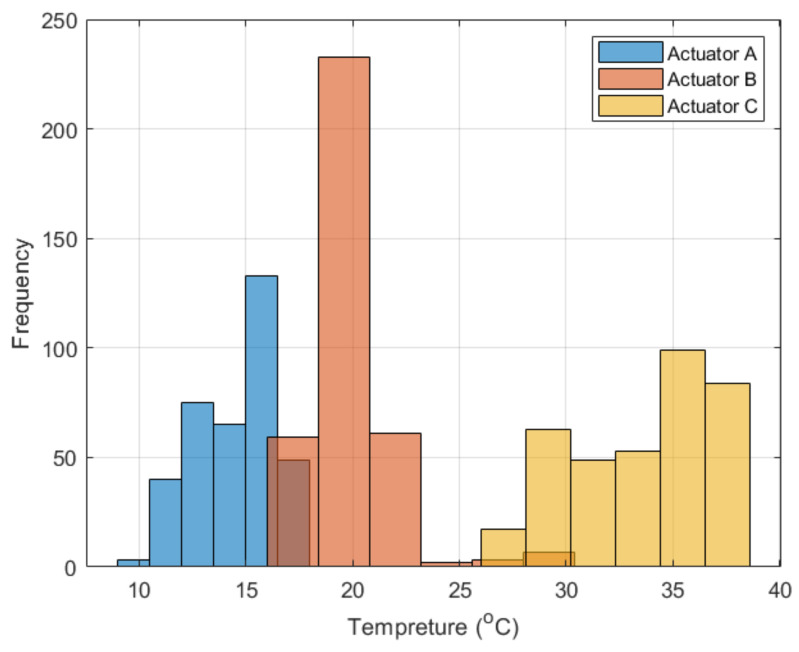
Histogram plot for the daily temperature variation of the three actuator designs.

The daily temperature variation is the mathematical difference between the highest and the lowest temperature of the SHC in a single day. This analytical data is crucial as it directly infers about the possible activating and deactivating ranges for the SMA-based actuator. It indeed permits daily activation and deactivation of the actuation mechanism due to the extensive range of temperature spans for substantial daily temperature variation. Moreover, Figure 13 shows the histogram plot of the daily temperature variation for the three proposed actuators. Actuators A, B, and C recorded an average daily temperature variation of 14.62, 19.83, and 33.65 °C, respectively. Therefore, it has been found through the conducted analysis that the optimized actuator, actuator C, is the most suitable actuator for the intended self-cleaning application, where it recorded an average temperature variation of 130.16% and 69.69% compared to actuators A and B, respectively.

**Figure 14:**
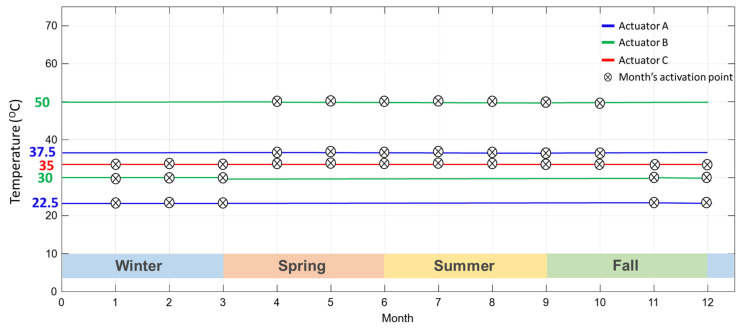
Activation temperature lines for the proposed three actuators.

The proposed PV cleaning system depends entirely on the thermal energy delivered to the SMA springs by solar radiation. An activation temperature lines term has been introduced here, which shows the temperature at which the SMA springs are anticipated to activate in particular months. Figure 14 presents these activation temperature lines for the proposed three actuators. The graph indicates that actuators A and B require a minimum of two activation temperature lines; hence, two sets of SMA springs are needed to operate independently throughout the year. In addition, actuators A and B have dedicated temperature activation lines for warm weather and another for cold weather. The fact that both actuators require two activation temperature lines led to the understanding that each of these actuators would need at least two maintenance routines per year for practical implementation. On the other hand, actuator C requires a single activation temperature line throughout the year in which minimum yearly maintenance routine is needed for its operation.

The comprehensive thermal studies for the different actuator designs concluded that actuator C is effective for the self-cleaning application. This valuable finding is supported by the outcomes of the conducted numerical study, where this actuator design has the most extensive daily temperature variation and can function throughout the year without needing maintenance.

### 3.3. Dynamic Appraisal of the Thermomechanical Actuator

Since the thermal investigation successfully identified an adequate design, the mechanical performance of this novel thermomechanical actuator is also considered. This section highlights multiple tests conducted to ensure the feasibility of utilizing the thermomechanical SMA solar-driven actuator as the actuation mechanism for the cleaning system from a mechanical energy perspective. The experimental tests evaluated the life expectancy of the NiTiNOL springs integrated into the actuation mechanism. They also assessed the actuation mechanism’s force and displacement.

**Figure 15:**
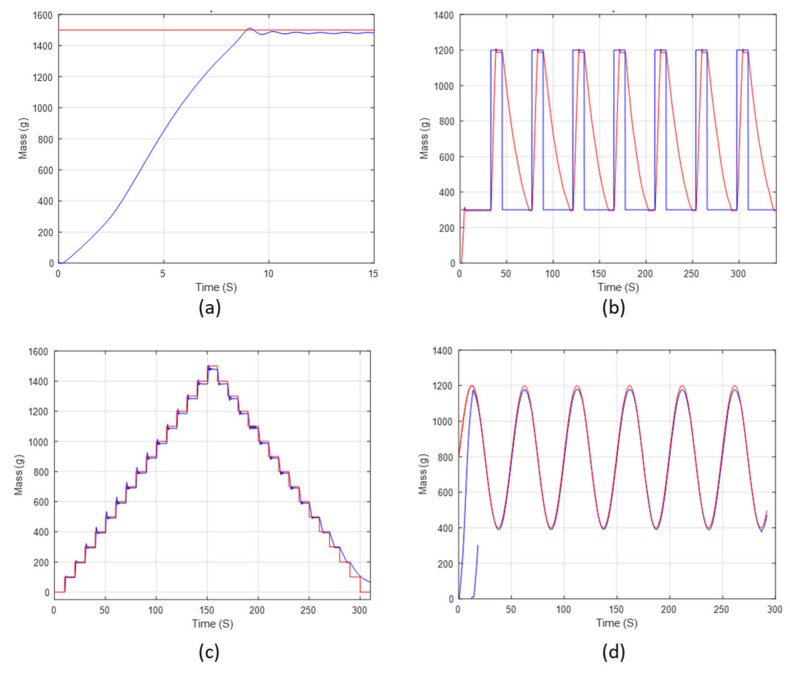
Gain scheduling PID controller’s Response to (**a**) step, (**b**) square-wave, (**c**) staircase, and (**d**) sinusoidal-wave command signals.

In order to carry out an expedited life expectancy test for the NiTiNOL spring actuator, a gain-scheduling PID controller with an anti-windup mechanism was implemented for the active actuation mechanism. In addition, multiple commands were used to ensure the controller’s usability, such as step, square, staircase, and sinusoidal command signals, as shown in Figure 15. the responses show the great accuracy of the controlled system, which enables further investigation of the expected life expectancy test.

**Figure 16:**
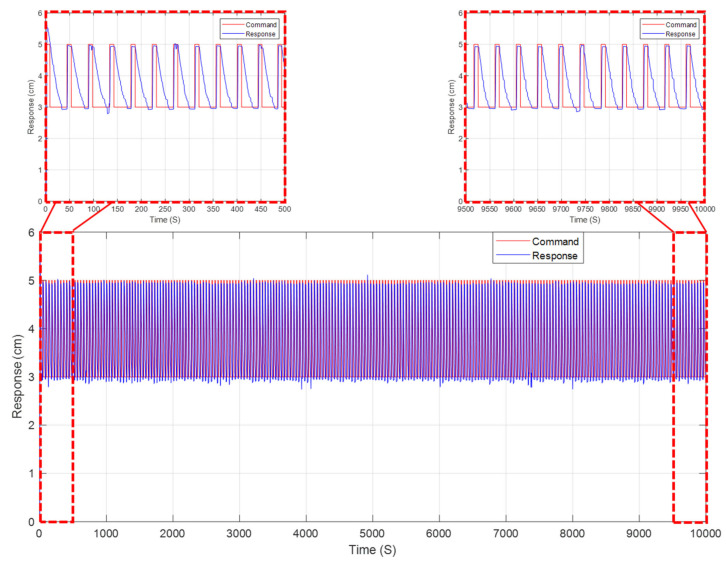
Long-duration displacement response of a square wave command signal.

For the life expectancy test, a long-duration square wave command signal was selected to rapidly simulate the force and displacement responses of the NiTiNOL spring in response to an applied heat source. Figure 16 shows, for instance, an example of the square wave used in both the displacement and force life expectancy tests. The NiTiNOL spring actuator used in the test handled over 7900 cycles of varying force between 2.95 N and 11.8 N, corresponding to about 20% and 80% of the maximum expected force. As for the displacement test, the total number of cycles ran was over 9200 cycles of varying displacement of 30 mm and 50 mm, corresponding to about 40% and 70% of the maximum displacement of the NiTiNOL spring. It can be concluded from the results of the life expectancy tests that the operation of the self-cleaning system daily can last over 20 years in service, which is approximately the life expectancy of conventional PV modules. The fact that the life expectancy of the actuator is closely similar to that of a conventional PV module shows the compatibility of the introduced self-cleaning mechanism and the PV module for the actual implementation of smart solar applications.

**Figure 17:**
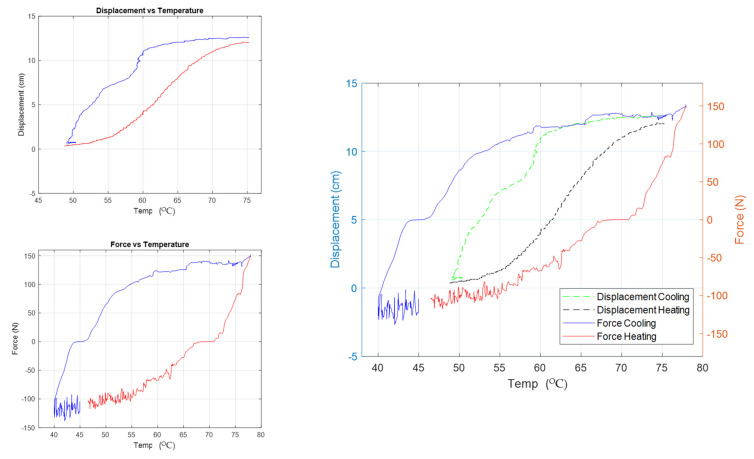
Displacement and force vs. temperature (hysteresis behavior).

Consequently, the force and displacement of the full-scale actuation mechanism were also tested to ensure that it can act as the actuation mechanism for the cleaning system. Figure 17 shows the hysteresis behavior of force, displacement, and a combined-case plots for the actuator. The carried-out results of the force test showed that the maximum pushing force was about 152.3 N at a temperature of 70.5 °C, while the maximum pulling force was 151.1 N. It can be found that a maximum bidirectional force of about 150 N can be produced. As for the maximum displacement, a stroke of about 127 mm at a temperature of 70.4 °C was recorded. Both force and displacement responses were plotted vs. temperature to extract the hysteresis behavior of the actuator, in which the mechanical work of the actuation mechanism was estimated of about 22.5 J.

The overall results of the dynamic appraisal have shown that the NiTiNOL springs used to convert the thermal energy into mechanical energy have a life expectancy of over 20 years, matching the life expectance of conventional PV modules. Additionally, experimental tests for the dynamic performance of the actuator showed that a bidirectional force over 150 N and a displacement of 127 mm could be produced, which achieves the desired design criteria for cleaning PV system.

### 3.4. Testing of the PV Cleaning System

**Figure 18:**
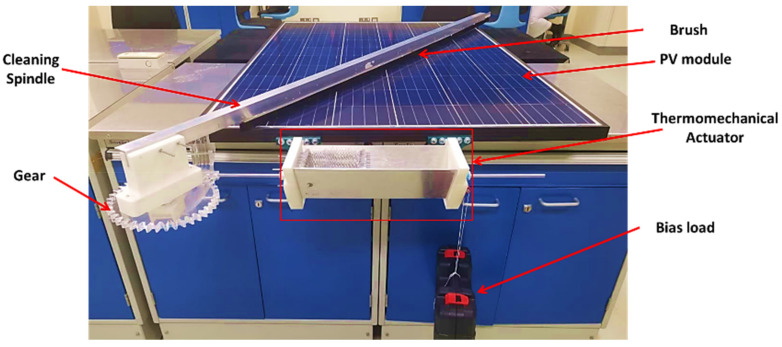
Actual setup for the smart PV cleaning system.

The smart cleaning system has been manufactured and evaluated for a single full-scale PV module of 250 W, as seen in Figure 18. Multiple indoor experiments were carried out to test the effectiveness of the smart cleaning system, where a heat source of an applied hot air device was applied to the fabricated thermomechanical actuator. As a result, it was found that the generated movement of the actuation mechanism enabled the rotation of the cleaning spindle over the PV module surface to remove the accumulated dust particles as designed successfully.

**Figure 19:**
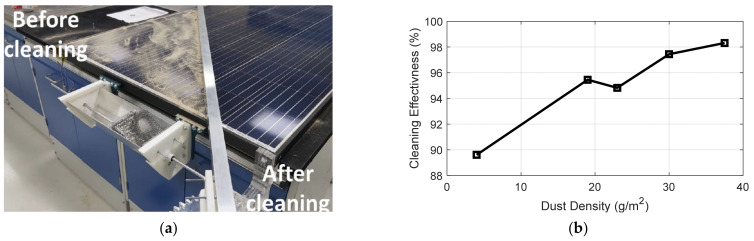
The cleaning effectiveness of the presented SMA-driven cleaning method under different dust densities, where (**a**) the actual performance of the self-cleaning operations, and (**b**) the quantitative outcome of the cleaning effectiveness percentages versus dust densities.

Furthermore, different amounts of dust were placed over the PV module to study the cleaning effectiveness based on different dust densities. The cleaning effectiveness test showed a promising result, as seen in Figure 19a, compared to the cleaning effectiveness of various cleaning methods mentioned in the literature. The average cleaning effectiveness of the cleaning system under different dust densities was computed from the data highlighted in Figure 19b to be 95.14%. As presented in the figure, the dust density over a PV module increases and the cleaning effectiveness increases, showing a direct correlation. The direct relation between the dust density and the cleaning effectiveness led to the conclusion that the cleaning method presented is more effective after sandstorms because additional dust particles accumulate over the surface of PV modules during such environmental and harsh weather conditions. Thus, periodic cleaning of PV modules leads to continuous power generation and minimum power losses due to dust particles accumulating over the surface, which is dominant in high dust density regions such as the MENA region.

The outcomes show that the developed actuation mechanism can function throughout the year with minimum maintenance process for its operation. Additionally, the similar expected life expectancy of the SMA-based cleaning actuator compared to conventional PV modules adds enormous benefits for its real-life implementation with modern PV applications.

## 4. Conclusions

To conclude, the presented paper discusses the design and fabrication of a novel passive autonomous smart cleaning system to solve the dust accumulation issue facing PV systems around the globe, particularly in the MENA region. Multiple tests were conducted, including thermal analysis of different designs of a thermomechanical SMA actuator, its mechanical assessment, and cleaning effectiveness of the proposed innovative self-cleaning solution, which assure the promising feasibility and real-life applicability. It can be briefly summarized that this study presents:

- Solar-powered and effective PV cleaning systems are a first-of-its-kind novel approach to overcome the dust accumulation issue facing PV systems.
- Thermal analysis results prove that the standalone cleaning system can operate under actual weather conditions in the MENA region, Dammam city.
- The optimized design of the developed SHC shows a minimum maintenance routine required for a continuous full-year operation.
- A solar-based bidirectional actuator with a stroke of about 126 mm and maximum push and pull forces of about 152.3 N and 151.1 N, respectively, were successfully achieved.
- The actuator’s thermal to mechanical conversion efficiency was recorded to be 19.15%, whereas the self-cleaning system has average cleaning effectiveness of 95%.

Future work could consider further thermal analyses of the novel cleaning system for several areas with different weather conditions to assess the feasibility of the design in different environments and worldwide. Furthermore, outdoor experiments are recommended to evaluate the designed cleaning system under real-world conditions. Lastly, a comprehensive energy comparison between a naturally cleaned PV module and a PV module equipped with the self-cleaning system is encouraged for further assessment and cost analysis.

## Figures and Tables

**Table 1:** FEA results of all developed parts within the mechanical system.

Part	Displacement (mm)	Strain	Stress (N/m^2^)	Factor of Safety
Actuator A Holder	2.92	5.67 × 10^−3^	1.7 × 10^7^	2.29
Actuator B Holder	4.12	8.12× 10^−3^	2.73 × 10^7^	1.43
Actuator C Holder	5.1	1.02 × 10^−2^	3.09 × 10^7^	1.26
Pinion	2.08 × 10^−2^	3.77 × 10^−4^	1.35 × 10^6^	33.33
Rack	5.52 × 10^−2^	6.47 × 10^−4^	2.31 × 10^6^	19.5
Spindle holder	1.09 × 10^−1^	7.88 × 10^−4^	2.76 × 10^6^	1.41

**Table 2:** Statistical data for the temperature inside the SHC over one year.

	Actuator A (°C)	Actuator B (°C)	Actuator C (°C)
Max	49.61	62.1	68.35
Min	13.58	13.5	11.2
Mean	31.03	39.7	35.05
Median	31.2	40	32.2
Range	36.03	48.5	57.15
Standard Deviation	9.34	10.8	15.16
